# Outbreaks of Short-Incubation Ocular and Respiratory Illness Following Exposure to Indoor Swimming Pools

**DOI:** 10.1289/ehp.9555

**Published:** 2006-11-28

**Authors:** Anna B. Bowen, James C. Kile, Charles Otto, Neely Kazerouni, Connie Austin, Benjamin C. Blount, Hong-Nei Wong, Michael J. Beach, Alicia M. Fry

**Affiliations:** 1 Enteric Diseases Epidemiology Branch, National Center for Zoonotic, Vector-borne and Enteric Disease; 2 Epidemic Intelligence Service, Office of Workforce and Career Development; 3 Environmental Health Services Branch, National Center for Environmental Health and; 4 Air Pollution and Respiratory Health Branch, National Center for Environmental Health, Centers for Disease Control and Prevention, Atlanta, Georgia, USA; 5 Illinois Department of Public Health, Springfield, Illinois, USA; 6 Volatile Organic Compound Laboratory, National Center for Environmental Health and; 7 Division of Parasitic Diseases, National Center for Infectious Disease, Centers for Disease Control and Prevention, Atlanta, Georgia, USA

**Keywords:** chloramines, cyanuric acid, disease outbreaks, indoor air pollution, swimming pools, trihalomethanes

## Abstract

**Objectives:**

Chlorination destroys pathogens in swimming pool water, but by-products of chlorination can cause human illness. We investigated outbreaks of ocular and respiratory symptoms associated with chlorinated indoor swimming pools at two hotels.

**Measurements:**

We interviewed registered guests and companions who stayed at hotels X and Y within 2 days of outbreak onset. We performed bivariate and stratified analyses, calculated relative risks (RR), and conducted environmental investigations of indoor pool areas.

**Results:**

Of 77 guests at hotel X, 47 (61%) completed questionnaires. Among persons exposed to the indoor pool area, 22 (71%) of 31 developed ocular symptoms [RR = 24; 95% confidence interval (CI), 1.5–370], and 14 (45%) developed respiratory symptoms (RR = 6.8; 95% CI, 1.0–47) with a median duration of 10 hr (0.25–24 hr). We interviewed 30 (39%) of 77 registered persons and 59 unregistered companions at hotel Y. Among persons exposed to the indoor pool area, 41 (59%) of 69 developed ocular symptoms (RR = 24; 95% CI, 1.5–370), and 28 (41%) developed respiratory symptoms (RR = 17; 95% CI, 1.1–260) with a median duration of 2.5 hr (2 min–14 days). Four persons sought medical care. During the outbreak, the hotel X’s ventilation system malfunctioned. Appropriate water and air samples were not available for laboratory analysis.

**Conclusions and relevance to professional practice:**

Indoor pool areas were associated with illness in these outbreaks. A large proportion of bathers were affected; symptoms were consistent with chloramine exposure and were sometimes severe. Improved staff training, pool maintenance, and pool area ventilation could prevent future outbreaks.

Swimming is a popular activity and sport among people of all ages. Outbreaks of illness caused by infectious organisms associated with swimming pool use are reported with regularity (Yoder et al. 2004). Proper pool maintenance, especially appropriate water disinfection procedures, and monitoring of pool water quality are key factors in the prevention of infectious diseases associated with use of swimming pools (Nemery et al. 2002). However, although chlorine kills many potential pathogens, it can also react with human wastes such as perspiration, urine, skin particles, and lotion in pool water to form chloramines and trihalomethanes. Chloramines may remain in the pool water or volatilize into the air, where they create the pungent smell and acutely irritating properties of air above swimming pools (Aggazzotti et al. 1990; Goyder 2000; Hery et al. 1995).

Chloramines have been suspected as a cause of occupational asthma and pneumonitis among lifeguards (Massin et al. 1998; Nemery et al. 2002; Thickett et al. 2002). Also, an outbreak associated with a swimming pool and attributed to chloramine exposure led to cough or ocular symptoms in > 70% of pool patrons interviewed (Goyder 2000). Chloramine levels in pool water depend on chlorine and nitrogen concentrations, pool pH, temperature, and water circulation patterns (Hery et al. 1995; Massin et al. 1998; Nemery et al. 2002). However, chloramine levels in the air above swimming pools are also influenced by ventilation and the pool water chemistry (Hery et al. 1995; Massin et al. 1998). Indoor pools are likely to be less well ventilated than outdoor pools, so the risks associated with chloramine exposure are likely to differ between indoor and outdoor swimming pools.

We investigated two outbreaks of acute ocular and respiratory symptoms associated with exposure to indoor swimming pools. In January 2004, the Illinois Department of Public Health was notified of two outbreaks of acute ocular and respiratory symptoms associated with indoor swimming pool exposure among patrons of two hotels located in noncontiguous central Illinois counties. We investigated these outbreaks during January 2004 to describe illness syndromes, determine risk factors for illness, and develop recommendations to prevent future incidents.

## Methods

We conducted retrospective cohort studies among guests of hotels X and Y. Hotel X staff routinely recorded all guests in the hotel registry; only one guest per room was recorded in the hotel Y registry. Study participants included registered persons at hotel X and both registered and unregistered persons at hotel Y who stayed at the hotels during the night before or after the date the respective outbreak began. Adult participants were interviewed by telephone with a standardized questionnaire following informed consent; children were interviewed with the assistance of a parent following assent. Information about underlying medical conditions, symptoms of illness, and exposure to all common areas in the respective hotel was collected. Participants were asked to estimate the timing of their exposures to the nearest minute. Interviews were conducted within 2.5 weeks of the outbreak at hotel X and within 1 week of the outbreak at hotel Y.

We defined an ocular case as any individual who developed at least one of the following after arrival at the hotel: burning eyes, watery eyes, blurry vision, difficulty opening eyes, or photophobia. A respiratory case was defined as an individual who developed at least one of the following symptoms after arrival at the hotel: cough, wheezing, shortness of breath, chest tightness, or sore throat. We performed bivariate and stratified analyses using chi-square, 2-tailed Fisher’s exact, and Cochran-Mantel-Haenszel tests; *p*-values < 0.05 were considered statistically significant. We also calculated the relative risks (RR) and 95% confidence intervals (CIs). The effect of duration of pool exposure was modeled for either syndrome at both hotels using a smoothing spline fit with λ = 100,000 (JMP 5.1.2; SAS Institute Inc.; Cary, NC).

Environmental health investigations were conducted at both hotels using an extensive environmental health outbreak investigation report for swimming pools [Centers for Disease Control and Prevention (CDC) 2006]. We examined swimming pool and spa dimensions, and disinfection, filtration, and ventilation systems; we also identified chemicals in the mechanical room of each pool. We collected pesticide application and pool maintenance records for the 2 weeks preceding the outbreak and interviewed swimming pool staff about recent pool maintenance practices and changes in equipment or building structure. No air samples were collected during the outbreak. Water samples were collected by the state health department for microbial analysis at hotel X but were discarded before chemical analysis. A water sample collected in a plastic drinking water bottle by a hotel guest approximately 12 hr after the outbreak at hotel Y was tested with gas chromatography and mass spectrometry for four trihalomethanes (chloroform, bromoform, bromodichloromethane, and dibromochloromethane) using a method similar to that of Cardinali et al. (2004); it was not tested for chloramines. No clinical specimens were available.

## Results

### *Hotel X.* Cohort study

Questionnaires were completed by 46 (60%) of the 77 guests at hotel X during the outbreak period. The most common symptoms included burning eyes, watery eyes, cough, sore throat, and headache (Table 1). A total of 26/31 (84%) persons with pool area exposure became ill: 22 met the case definition for the ocular syndrome and 15 for the respiratory syndrome (Table 1). Demographics were similar between the groups with either syndrome (Table 2). One child sought medical care for respiratory symptoms.

Neither syndrome was associated with sex, an underlying diagnosis of asthma or environmental allergies, exposure to tobacco smoke within the home, or use of contact lenses.

**Figure 1:**
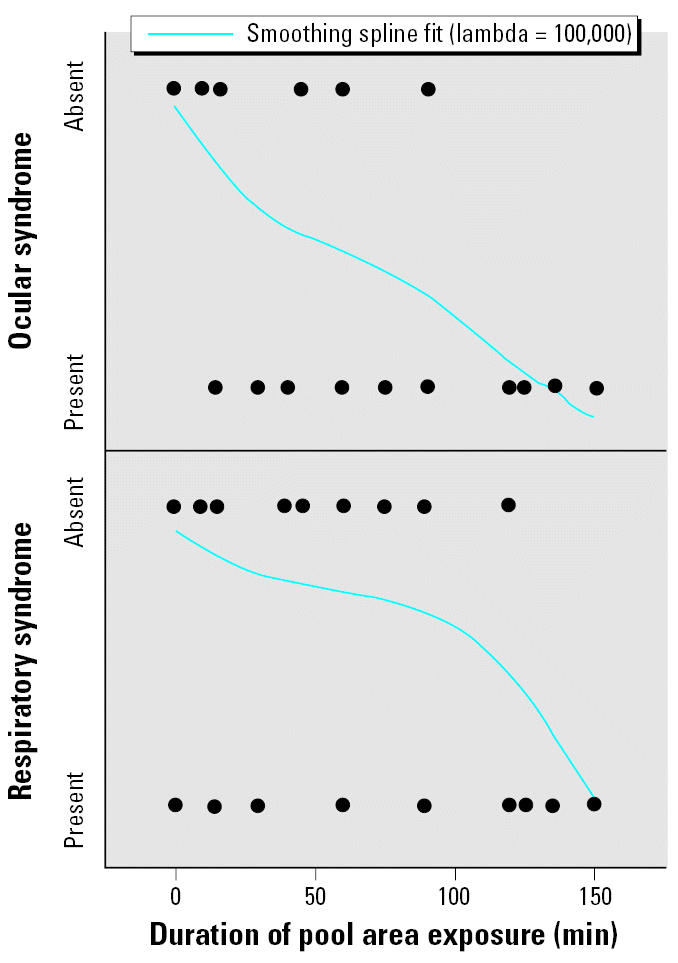
Effect of duration of swimming pool area exposure on risk of illness at hotel X. For the ocular syndrome, *R*^2^ = 0.62; sum of squares error = 4.42. For the respiratory syndrome, *R*^2^ = 0.35; sum of squares error = 6.63.

Thirty-one people (66%) entered the pool area, and 20 (43%) entered the swimming pool. Exposure to the swimming pool area, swimming pool, and whirlpool were associated with both ocular and respiratory illnesses (Table 3). Exposure to the pool area without pool or whirlpool contact was associated with the ocular syndrome; age < 18 years was associated with the respiratory syndrome. All of the people who met the ocular case definition and 14 (93%) who met the respiratory case definition entered the pool area. Among those who entered the swimming pool, activities such as jumping, splashing, submerging one’s head underwater, or swallowing water were not additional risk factors for illness; use of swimming goggles did not protect against the ocular syndrome. Length of exposure to the swimming pool area correlated with either syndrome (Figure 1). The median incubation time between pool area exposure and onset of either syndrome was 1.2 min (range, 0–9 min). The median duration was 1.75 hr (range, 5 min–5 hr) for ocular symptoms and 2.5 hr (range, 2 min–14 days) for respiratory symptoms.

### Environmental health investigation

The outbreak was associated with a 9,500 gallon pool and an 88-inch diameter whirlpool housed within feet of each other inside hotel X. The pool was tended by a single maintenance person each weekday and by another staff person the remaining 2 days of the week; the outbreak began on a weekend when usual maintenance staff were absent. The pool and whirlpool were fed by separate pump and filter systems. A stabilized chlorine disinfectant product, trichlors-triazinetrione, commonly sold as combination chlorine/cyanuric acid tablets for use in outdoor pools, was used in both the pool and whirlpool automatic feeders. The water used for filling the pool and spa was chloraminated at the municipal water treatment center.

Pool maintenance logs revealed elevated free chlorine levels in the swimming pool during the outbreak. The whirlpool free chlorine level could not be quantified because it exceeded the scale of the test kit being used, and staff did not perform dilutions to obtain an accurate measurement. On the day after the outbreak, the environmental health employees from the state health department measured swimming pool and whirlpool chemical abnormalities (Table 4). This investigation also found that the recirculation systems and the automatic disinfection systems appeared to be functioning correctly. The swimming pool water sample taken by the state health department did not yield coliforms, *Pseudomonas, Cryptosporidium*, or *Giardia*.

The pool area ventilation system had been malfunctioning for several days, so the heating, ventilation, and air-conditioning system was being regulated manually at the time of the outbreak. Because of the low temperatures during January in Illinois, no natural ventilation was being used.

### *Hotel Y.* Cohort study

Thirty (39%) of 77 registered guests and 58 of their companions at hotel Y were reached, yielding 88 completed questionnaires. Forty-one people met the case definition for the ocular syndrome and 28 the respiratory syndrome (Table 2). Of 69 persons with pool exposure at hotel Y, 46 (72%) became ill. Two people were seen in a hospital emergency department and 1 by a private physician as a result of these symptoms.

Illness was not associated with sex, an underlying diagnosis of asthma or environmental allergies, exposure to tobacco smoke within the home, or use of contact lenses.

**Figure 2:**
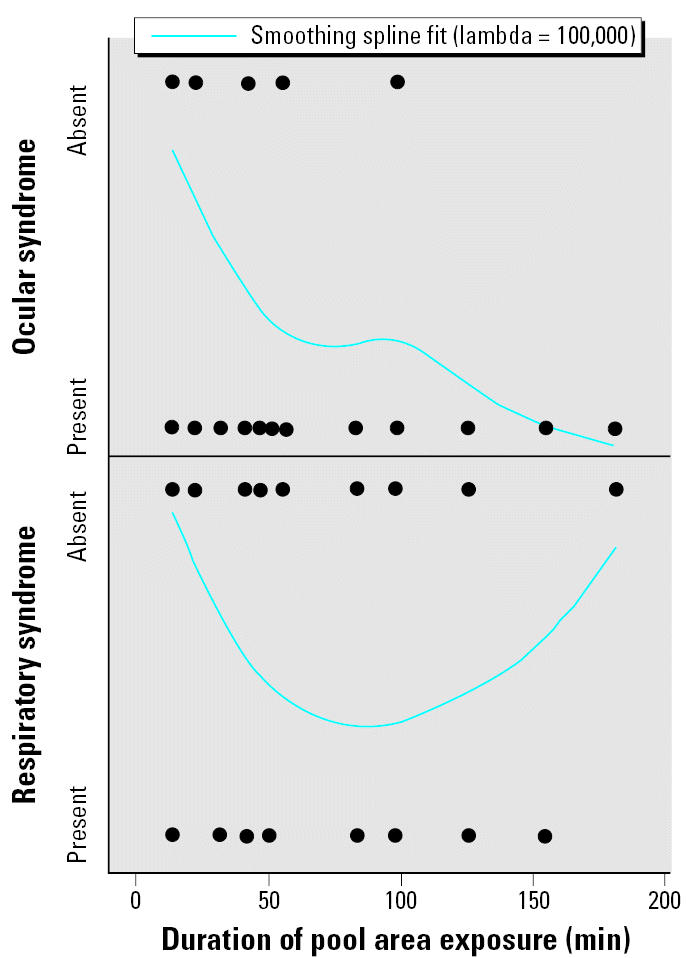
Effect of duration of swimming pool area exposure on risk of illness at hotel Y. For the ocular syndrome, *R*^2^ = 0.36; sum of squares error = 14.01. For the respiratory syndrome, *R*^2^ = 0.35; sum of squares error = 12.3.

Sixty-nine people (78%) entered the swimming pool area, and 46 (52%) entered the swimming pool. Exposure to the swimming pool area or swimming pool as well as age < 18 years were associated with illness (Table 5). Everyone who met either case definition entered the pool area. Activities within the pool that increased immersion, such as dunking head underwater, were not associated with additional risk for illness, and use of swimming goggles did not protect against the ocular syndrome. Longer duration of exposure to the swimming pool area was associated with the ocular syndrome; increasing duration of exposure to the swimming pool area up to 1 hr correlated with development of the respiratory syndrome, but thereafter, duration of exposure was inversely associated with respiratory illness (Figure 2). The median incubation time between pool area exposure and onset of symptoms was 1.2 min (range, 0–17 min) for the ocular syndrome and 7 min (range, 1.2–59 min) for the respiratory syndrome. The median duration was 1.5 hr (range, 5 min–24 hr) for ocular symptoms and 10 hr (range, 15 min–24 hr) for respiratory symptoms.

### Environmental health investigation

The outbreak was associated with a 13,900 gallon pool inside hotel Y and began on a weekend. Maintenance was performed as usual by the hotel manager on the day the outbreak began. Trichlors-triazinetrione was used for disinfection; the tablet brand differed from that used in hotel X. Maintenance records revealed twice-daily chemistry checks, weekly combined chlorine measurements, and near-weekly cyanuric acid concentration measurements. The municipal water used to fill the pool was chlorinated. We did not find any staff or guests associated with both hotel X and hotel Y.

On the day the outbreak began, maintenance records indicated an elevated combined chlorine level of 1.5 (normal < 0.5) ppm within the pool; free chlorine and cyanuric acid levels, pH, and temperature were in the expected range. According to the hotel manager, approximately 60–70 children entered the pool that evening, exceeding the 41-bather limit. The pool manager used approximately 13 ounces of 67% calcium hypochlorite to hyperchlorinate the pool when it closed for the evening.

During an inspection by the state health department the following day, combined chlorine and cyanuric acid levels were elevated (Table 4). The circulation, disinfection, and ventilation systems appeared to be functioning properly.

Trihalomethane levels found in these samples were within the range normally measured in U.S. indoor pools (3–580 ng/mL) (Armstrong and Golden 1986).

## Discussion

A total of 72 people became ill with ocular and respiratory illness during outbreaks associated with indoor swimming pools at two hotels. We observed symptoms similar to those previously reported following chloramine exposure, including burning eyes, tearing, and cough (Goyder 2000; Massin et al. 1998; Momas et al. 1993; Thickett et al. 2002; Varraso et al. 2002). Symptoms occurred within minutes of exposure to indoor swimming pool environments, generally were more likely to develop with increased duration of exposure, and were sometimes prolonged and severe. We were able to document illnesses lasting up to 14 days; four bathers also sought medical care as a result of these outbreaks. As in a previous outbreak, children were predominantly affected, and attack rates were extremely high (Goyder 2000).

In the outbreaks at hotel X and hotel Y, exposure to the pool area was associated with symptoms; in at least some cases, contact with the pool or whirlpool water was not required. The ocular syndrome developed only among persons exposed to the pool area; the respiratory syndrome developed in 1 of 15 persons without pool-area exposure at hotel X but in no one without pool-area exposure at hotel Y. Activities within the pool, such as swimming under water or swallowing water, were not additional risk factors for illness, and use of swimming goggles within the pool area was not associated with lower risk of the ocular syndrome. This suggests that contact with or inhalation of contaminated air alone is associated with symptoms—as is expected with a mucosal irritant—and that dermal absorption or ingestion may not be necessary to produce symptoms. Generally, longer exposure to the pool area correlated with illness. However, those who remained longest in the pool area of hotel Y developed ocular, but not respiratory, symptoms; the reasons for this are unclear.

Although the etiologic agent in these outbreaks could not be determined definitively, chloramines likely contributed to the illness. Levels of chlorine at the hotel X whirlpool likely exceeded chlorine-to-nitrogen mass ratios required for breakpoint chlorination and would have increased production of trichloramine (Shang and Blatchley 1999; White 1999). This, when combined with a malfunctioning ventilation system in the pool area and chloraminated fill water, could have led to elevated chloramine levels in the air around the pool. Similarly, although pool maintenance records did not indicate excess chlorine at the hotel Y pool, the bather load and combined chlorine levels exceeded recommended levels during the outbreak period. A sample of swimming pool water from hotel Y was analyzed by the CDC but did not reveal potential causal agents. Chloramines are highly volatile and would not be expected to persist under the given sample storage conditions; therefore, the water sample was not analyzed for chloramines. However, trihalomethanes, which are potential ocular and respiratory irritants that may be found in swimming pools (Shaw and Eng 1987), are chemically stable and therefore might persist at high levels if initial concentrations were substantial enough to offset loss from nonstandard sample collection and storage. This sample did not contain abnormally high levels of trihalomethanes compared with previously published levels for U.S. pools (Armstrong and Golden 1986). Thus, no other potential causal agents, such as microbial pathogens or elevated levels of trihalomethanes, were found in pool water from hotel Y. Additionally, the observed incubation period and symptoms at both hotels were typical of those observed following exposure to chloramines (Goyder 2000; Massin et al. 1998; Momas et al. 1993; Thickett et al. 2002; Varraso et al. 2002). Together, these findings suggest that chloramine exposure played a role in the outbreaks. To appropriately quantify environmental concentrations of chloramines, air or water sampling must take place within hours of identification of a possible outbreak and before changes have been made to pool chemistry or ventilation. Water samples should be collected in amber glass bottles, which might not be readily available. Air sampling and testing methods for chloramines are also complex and beyond the routine capability of most public health departments (Hery et al. 1995). It is likely that these factors will contribute to future underreporting of outbreaks of chloramine intoxication.

To decrease exposure to chloramines in swimming pool environments, proper ventilation is also required. Because of limited air turnover and the potential for continuous generation of chloramines, air quality in indoor swimming pool enclosures is likely to differ substantially from that of outdoor swimming pool areas. It is not surprising, therefore, that differing levels of chloramines between indoor and outdoor swimming pool environments have been reported (Massin et al. 1998). Special attention to pool chemistry and ventilation may be required in cold weather climates, where a greater proportion of air may be recycled to prevent heat losses, and in indoor recreational pools and parks where wave action, sprayers, and other turbulent water activities have been documented to increase the aerosolization and volatilization of chloramines to a greater degree than standard leisure pools (Hery et al. 1995). Because little or no data exist on airborne concentrations of chloramines for pools, the American Society of Heating, Refrigerating and Air-Conditioning Engineers (2006) is investigating whether the amount of ventilation air currently required in pool enclosures is sufficient to remove these pollutants.

To ensure adequate pool operation and ventilation, it is critical to improve the training of pool operators. Currently, pool staff commonly lack adequate training in swimming pool chemistry and maintenance (CDC 2003). Maintenance staff also may be required to divide their time between oversight of the pools and many other activities, as in these two outbreaks. Violations of pool codes, especially swimming pool chemistry regulations, are a common result. In a study of > 22,000 pool inspections in 2002, more than half of inspections reported at least one violation. Child wading pools (18.4%), therapy pools (14.3%), and hotel/motel pools (14.0%) yielded the greatest percentage of violations (CDC 2003). In both outbreaks reported in the present study, pool chemistry abnormalities included elevated free and combined chlorine, as well as elevated cyanuric acid levels. Cyanuric acid is intended to protect chlorine from degradation due to ultraviolet light in outdoor swimming pools (Moody et al. 1993; Yamashita et al. 1988). As a result, higher free chlorine residuals are required in swimming pools treated with cyanuric acid (Yamashita et al. 1988), and it should be used only in outdoor swimming pools and spas. These problems suggest that standardized education of public pool operators should be mandatory. Additionally, trained personnel should be available for monitoring and maintenance duties at all times, especially at times when pool usage is highest. Both of these outbreaks began on weekends when the bather load was high; in one outbreak (hotel X), pool maintenance was not performed by the usual staff. Clearly labeling pool products containing cyanuric acid for outdoor use may also decrease inappropriate use of these products.

There are several limitations to these studies. Although we attempted to reach every guest registered at the hotel during the outbreak period, participation was incomplete and reported attack rates may be artificially elevated. Participants also may not have been able to recall precisely their symptoms or exposures. Finally, the lack of appropriate air and water samples from the outbreak period limited our ability to establish a definitive etiology for the illnesses associated with these outbreaks.

Clinicians and public health practitioners should watch vigilantly for possible outbreaks of illness due to swimming pool exposure, especially during the indoor swimming pool season. When an outbreak is suspected, the local health department should be notified immediately and environmental health staff should investigate promptly. To help establish outbreak etiology, swimming pool or spa water chemistry (free and combined chlorine levels) should be immediately documented before alteration of the water chemistry, and appropriate sample dilutions should be made if readings exceed the limits of test kits. Development of simple, rapid, field-based methods for measurement of chloramines and indoor air quality for use by public health departments would assist outbreak investigations.

These outbreaks involved extremely high attack rates and short incubation periods following exposure to indoor swimming pool enclosures. Similar outbreaks are likely to be common, although they have been reported rarely. As indoor pool swimming continues to gain popularity, careful attention to pool maintenance should help prevent illness from infectious and chemical agents. Health departments should be prepared to investigate outbreaks within hours of their identification and collect appropriate environmental samples. The public should be alerted to the potential health risks of poor hygiene practices such as urinating in or not bathing before entering swimming pools.

## Figures and Tables

**Table 1:** Prevalence and duration of symptoms experienced by guests with swimming pool area exposure in hotels X (*n* = 31) and Y (*n* = 69).^a^

			Duration [median (range)]	
Symptom	Hotel X *n* (%)	Hotel Y *n* (%)	Hotel X	Hotel Y
Ocular	22 (71)	41 (59)		
Burning eyes	20 (65)	37 (54)	2 hr (5 min–9.5 hr)	2 hr (15 min–24 hr)
Watery eyes	10 (32)	15 (22)	1 hr (5 min–3 hr)	45 m (5 min–11 hr)
Trouble opening eyes	1 (3)	10 (14)	5 min	1 hr (15 min–12 hr)
Blurry vision	0 (0)	3 (4)	—	7.8 hr (4.5 hr–11 hr)
Photophobia	0 (0)	7 (10)	—	4.5 hr (1 hr–11 hr)
Respiratory	14 (45)	28 (41)		
Sore throat	6 (19)	14 (20)	16 hr (5 min–48 hr)	10 hr (4.5 hr–48 hr)
Dry mouth	3 (10)	4 (6)	13.5 hr (3 hr–24 hr)	17.5 hr (1 hr–34 hr)
Cough	11 (35)	26 (38)	2.5 hr (2 min–14 days)	12 hr (1.25 hr–2 days)
Wheezing	1 (3)	3 (4)	72 hr	17 hr (10 hr–24 hr)
Chest tightness	4 (13)	5 (7)	48 hr (2.3 hr–14 days)	7.8 hr (15 min–24 hr)
Other
Headache	5 (16)	4 (6)	2 hr (25 min–24 hr)	2.5 hr (1 hr–2 hr)
Burning nose	4 (13)	0 (0)	2.1 hr (30 min–24 hr)	—
Stuffy nose	3 (10)	5 (7)	16.8 hr (9.5 hr–24 hr)	8 hr (45 min–24 hr)
Sneezing	1 (3)	12 (17)	5 days	5 hr (20 min–13 hr)
Nausea	3 (10)	12 (17)	1.3 hr (35 min–2 hr)	2 hr (30 min–12 hr)
Vomiting	3 (10)	14 (20)	1 min (1 min–10 min)	—

a Less than 10% of respondents reported dizziness, abdominal cramps, diarrhea, fever, weakness, fatigue, or rash.

**Table 2:** Demographics of patients associated with hotels X and Y.

	Hotel	Ocular syndrome	Respiratory syndrome
No. male/total (%)	X	11/22 (50)	7/15 (47)
	Y	24/41 (59)	19/28 (68)
Median age [years (range)]	X	19 (2–58)	10 (6–57)
	Y	8.5 (6–55)	8 (3–42)
No. < 18 years of age/total (%)	X	11/22 (50)	10/15 (67)
	Y	32/41 (78)	25/28 (89)

**Table 3:** Risk factors for ocular or respiratory syndromes among guests at hotel X.

	Exposed		Unexposed			
Syndrome, exposure	Ill	Well	Ill	Well	RR (95% CI)	*p*-Value^a^
Ocular						
Entered pool area	22	9	0	15	24^b^ (1.5–370)	< 0.0001
Entered pool area only (no water contact)	6	2	0	16	25^b^ (1.6–390)	0.0002
Entered swimming pool	15	5	7	19	2.8 (1.4–5.5)	0.003
Entered whirlpool	14	7	8	17	2.1 (1.1–4.0)	0.04
Entered lobby	4	10	18	14	0.5 (0.2–1.2)	0.1
Entered dining area	21	20	1	4	2.6 (0.4–15)	0.3
Entered public restroom^c^	19	7	3	17	4.9 (1.7–14)	< 0.0001
Age < 18 years^d^	11	4	11	21	2.1 (1.2–3.8)	0.03
Respiratory						
Entered pool area	14	17	1	14	6.8 (1.0–47)	0.02
Entered pool area only (no water contact)	2	6	1	15	4.0 (0.42–38)	0.2
Entered swimming pool	12	8	3	23	5.2 (1.7–16)	0.001
Entered whirlpool	10	11	5	20	2.4 (1.0–5.9)	0.06
Entered lobby	5	9	10	22	1.1 (0.5–2.7)	1
Entered dining area	14	27	1	4	1.7 (0.3–10)	1
Entered public restroom^c^	13	13	2	18	5.0 (1.3–20)	0.005
Age < 18 years^d^	10	5	5	27	4.3 (1.8–10)	0.002

a Chi-square or Fisher’s exact *p*-value.

b Logit RR.

c Following stratification for pool area exposure, the public restroom was not significantly associated with either syndrome.

d After stratification for pool area exposure, age < 18 years was significantly associated with the respiratory but not the ocular syndrome.

**Table 4:** Environmental health findings at hotels X and Y.^a^

System	Hotel X	Hotel Y	Recommended range
Swimming pool			
Free chlorine (ppm)	7.5	2.0	1–4^b^
Combined chlorine (ppm)	< 0.5	1.0	< 0.5^b^
pH	7.4	7.5	7.2–7.6^b^
Temperature (°C)	28.9	29.4	24.4–33.3^b^
Cyanuric acid (ppm)	> 100	> 100	< 100
Whirlpool			
Free chlorine (ppm)	40	NA	1–4
Combined chlorine (ppm)	ND	NA	< 0.5
pH	ND	NA	7.2–7.6
Temperature (°C)	38.3	NA	~ 35–40
Recirculation system	Intact	Intact	Intact
Disinfection system	Intact	Intact	Intact
Ventilation system	Malfunctioning; regulated manually during outbreak	Intact	Intact

Abbreviations: NA, not applicable; ND, not determined.

a Samples tested by investigators 1 day after the outbreak.

b Illinois State pool code (Joint Committee on Administrative Rules 1999).

**Table 5:** Risk factors for ocular and respiratory syndromes among guests at hotel Y.

	Exposed		Unexposed			
Syndrome, exposure	Ill	Well	Ill	Well	RR (95% CI)	*p*-Value^a^
Ocular						
Entered pool area	41	28	0	19	24^b^ (1.5–370)	< 0.0001
Entered pool area only (no water contact)	6	16	1	19	5.5 (0.72–41)	0.1
Entered swimming pool	34	12	6	35	5.1 (2.4–11)	< 0.0001
Entered lobby	20	20	18	27	1.3 (0.8–2.0)	0.4
Entered dining area	13	14	25	33	1.1 (0.7–1.8)	0.8
Entered public restroom	7	6	31	41	1.3 (0.7–2.2)	0.6
Entered exercise room	0	1	34	46	0 (—)	1
Drank from drinking fountain^c^	8	2	22	41	2.3 (1.4–3.6)	0.01
Ate pizza	23	21	15	26	1.4 (0.9–2.3)	0.2
Age < 18 years^d^	32	12	7	32	4.1 (2.0–8.1)	< 0.0001
Respiratory						
Entered pool area	28	40	0	19	17^b^ (1.1–260)	0.0002
Entered pool area only (no water contact)	2	20	0	19	4.3^b^ (0.22–85)	0.5
Entered swimming pool	26	20	2	39	12 (2.9–46)	< 0.0001
Entered lobby	14	26	14	31	1.1 (0.6–2.1)	0.8
Entered dining area	10	17	18	40	1.2 (0.6–2.2)	0.6
Entered public restroom	4	9	24	48	0.9 (0.4–2.2)	1
Entered exercise room	0	1	24	56	0 (—)	1
Drank from drinking fountain^c^	7	3	13	50	3.4 (1.8–6.4)	0.003
Ate pizza	14	30	12	29	1.1 (0.6–2.1)	0.8
Age < 18 years^d^	25	19	3	36	7.4 (2.4–23)	< 0.0001

a Chi-square or Fisher’s exact *p*-value.

b Logit RR.

c Following stratification for pool area exposure, the drinking fountain was not significantly associated with either syndrome.

d After stratification for pool area exposure, age < 18 years remained significantly associated with both syndromes.
